# Editorial: Cancer Cell Metabolism and Immunomodulation in the Context of Tumor Metastasis

**DOI:** 10.3389/fonc.2021.803213

**Published:** 2022-02-10

**Authors:** Qiongzhu Dong, Peter J. Nelson, Yue Zhao

**Affiliations:** ^1^ Key Laboratory of Whole-Period Monitoring and Precise Intervention of Digestive Cancer, Shanghai Municipal Health Commission (SMHC), Minhang Hospital, Fudan University, Shanghai, China; ^2^ Medical Clinic and Policlinic IV, Ludwig-Maximilian-University (LMU), Munich, Germany; ^3^ General, Visceral and Cancer Surgery, University Hospital of Cologne, Cologne, Germany

**Keywords:** cancer metabolism, immunomodulation, microenvironment, tumor metastasis, immune evasion

Primary tumor growth and the tumor metastasis are strongly linked to the adaption of the tumor tissues to environmental issues. These can include growth in low oxygen conditions and the selective use of substances for energy needs as well as means to escape detection by the immune system. Metastasis represents the major cause for poor prognosis for patients with cancers (1). Cancer patients are diagnosed with metastatic disease either at diagnosis of the disease, or later as they emerge during the course of treatment (2). Metabolic alterations and immune evasion represent some of the hallmark capabilities of tumor cells that develop during multistep metastatic progression (3). Cancer cells become adapted to survive with an energy supply and additional growth stimuli imparted through the tissue environment during the metastatic process. At the same time, cancer cells evade destruction by the immune system through various adaptions that enables them to progress. Various immune cells and immune processes have been linked to both the inhibition, and promotion, of metastatic processes. Recent advances in immunotherapy and the development of metabolic oncology inhibitors are providing exciting new approaches for the treatment of cancer (4). As we are learning more about the molecular basis of cancer metabolism and the general biology of the tumor immune microenvironment, novel strategies are emerging that may help prevent or control cancer metastasis. Tumor immunity and metabolism in cancer metastasis was the goal of this Research Topic. The collection includes 29 original research papers and reviews directed toward the advancement of our understanding of these important topics in cancer research.

## Cancer Cell Metabolism in the Context of Tumor Metastasis

Cancer cells acquire the ability to remodel their metabolic network allowing them to adapt and maintain their survival in the context of extreme changes to their environment. Some of the key findings outlined in the reviews and original research papers contained in this special issue as detailed below. These observations provide insight into the emerging field of metabolic control during cancer metastasis. Wolfe et al. highlighted the concept of metabolic compartmentalization independent of the cell membrane, as a critical mechanism helping to determine tumor invasion and metastasis. The authors focus on the importance of compartmentalization of purine nucleotide metabolism (e.g., ATP and GTP) at the leading edge of migrating cancer cells. They describe uniquely phase-separated microdomains where a dynamic exchange of nucleotide metabolic enzymes takes place (Wolfe et al.). Dysregulation of the enzyme ALDH1A3 (aldehyde dehydrogenase 1 family member A3) was linked by Nie et al. to a highly aggressive pancreatic cancer subtype. They found that ALDH1A3 promoted pancreatic cancer metastasis *via* influence on glucose metabolism (Nie et al.). Guo et al. demonstrated that pyruvate kinase M2 (PKM2) expression was upregulated in prostate cancer and was positively associated with tumor metastasis. The molecular basis of PKM2-driven prostate cancer metastasis was linked to ERK-cyclooxygenase (COX-2) signaling (Guo et al.). Several studies identified key regulators involved in the metabolic adaptation required for metastasis including nuclear factor erythroid-2-related factor-2 (NFE2L2/Nrf2) and the Hippo signaling factors Yes-Associated Protein (YAP)/Transcriptional Co-activator with PDZ-binding Motif (TAZ). Zhao et al. summarized the molecular basis for the role of Nrf2-mediated metabolic reprogramming in non-small cell lung cancer and identified potential therapeutic targets in the network underlying Nrf2-mediated metabolic reprogramming. Yamaguchi and Taouk discussed the YAP/TAZ functions as a central hub for metastasis by regulating metabolic alterations.

Amino acid metabolism is deregulated in many cancers, with changes in amino acid metabolism specifically providing growth nutrients used by cancer cells (5). Zhao et al.’s team reviewed the mechanisms and the role of amino acid metabolism in regulating pancreatic cancer progression. Moreover, the authors discussed recent advances in therapeutic strategies directed toward targeting amino acid biology for the treatment of pancreatic cancer (Xu et al.). The regulation of glutaminase and its role in cancer metastasis was the focus of the study by Wang et al. This group discussed possible mechanisms of glutaminase inhibitor resistance and potential strategies that could be employed to help overcome the therapy resistance (Wang et al.).

The process of autophagy is emerging as an important process required for tumor metastasis and metabolism (6). Based on the expression autophagy-related genes (ARGs) taken from the MsigDB database, Chen et al. were able to divide ovarian cancer patients into two subtypes and were able to establish an expression signature for risk stratification that could be used to help predict tumor outcome and to better characterize the tumor immune microenvironment. The authors found that ULK2 and GABARAPL1 may play important roles in ovarian tumorigenesis and thus may represent immunotherapy targets in ovarian cancer (Chen et al.). Wen et al. demonstrated that SNHG9, a papillary thyroid cancer cell exosome-enriched lncRNA, inhibits cell autophagy and promotes cell apoptosis of normal thyroid epithelial cell Nthy-ori-3 through the YBOX3/P21 pathway.

## Immunomodulation in the Context of Tumor Metastasis

The long-sought promise of immune control of cancer has finally emerged in the past decade and now represents some of the most important new tools for control of cancer in the clinic (7). Several reviews and original research papers addressed new advancements in our understanding of the role of immunomodulation in the context of tumor metastasis and provided ideas for expanding clinical cancer therapy. A review by Ham et al. discussed a recent study on how cancer cells can influence monocyte-derived and tissue-resident macrophage polarization *in vivo*. Based on high-throughput transcriptomic data, Zeng et al. identified an immune cell infiltration pattern in pre-metastatic liver cancer and highlighted the potential importance of myeloid-derived suppressor cells (MDSCs) as a dominant altered cell type in colorectal cancer. Using two different types of experimental mouse cancers (melanoma and carcinoma), Bhuniya et al. showed that neem leaf glycoprotein (NLGP), a natural immunomodulator, helps to foster antigen-specific activated CD8+ T cells *via* altering the maturation of dendritic cells (DCs) to help combat metastasis.

Changes in cellular metabolism are also an important mechanism for the regulation of immunity that are associated with an impairment of the anti-tumor immune response. Hung et al.’s team discussed how tumor cells can reprogram tissue metabolism to help foster a pro-tumor microenvironment, driving disease progression and immune evasion. The authors highlighted potential approaches to help target metabolic vulnerabilities in the context of anti-tumor immunotherapy (Jiang et al.). Wu et al. reviewed mechanisms of immune escape caused by metabolic reprogramming, with an aim toward providing new targets for enhancing clinical tumor immunotherapy and overall tumor treatment through metabolic intervention. Mitochondrial activity is centrally important in the crosstalk between tumors and the immune system. Klein et al. discussed the role of mitochondrial action in cancer immune evasion and summarized the effects of mitochondria-targeted antitumor therapy. Zheng et al.’s group identified an important role for glutamine starvation in regulating expression of G-CSF and GM-CSF and thus in facilitating the generation of immunosuppressive MDSCs in the context of breast cancer. Iron metabolism has been linked to the regulation of tumor growth, metastasis and the general response to immunotherapies. Brown et al. reported on the dual role of iron metabolism including its regulation and storage during tumor progression and in control of the immune response. The authors further discussed the potential for iron-based therapeutic strategies in combination with other therapies, including immunotherapies for treating metastatic disease (Brown et al.).

Due to reduced oxygen levels in tumor tissues and the accompanying Warburg effect, production of lactic acid is enhanced in most tumors that is in turn correlated with tumor aggressiveness and poor clinical outcome. Fischbeck et al. reported that lactic acid can inhibit T cell killing activity by reducing cytotoxic T cell (CTL) motility, arrest and prolonging CTL contact duration with tumor cells, and through these events, reduce the overall effectiveness of the cytotoxic response. Lactic acid intervention and strategies to improve T cell metabolic fitness hold promise to help improve the clinical efficacy of T cell-based cancer immunotherapy (Fischbeck et al.). Pour et al. described the role of kynurenine metabolites in tumoral immune escape among patients with metastatic cutaneous malignant melanoma. They found that cutaneous metastatic melanoma can have high levels of altered kynurenine pathway associated gene expression and treatments with MAPK inhibitors are associated with changes in 3-hydroxykynurenine and 3hydroxyanthranilic acid (3HAA) concentrations that appears to lead to higher “CXCL11,” and “KLRD1” expression, factors involved in T and natural killer (NK) cell recruitment and activation (Pour et al.). Building upon the role of tumor immunotherapy as a treatment strategy, Qin et al.’s group found that PKM2 expression could drive hepatocellular carcinoma (HCC) progression by enhancing an immunosuppressive tumor microenvironment through PD-L1 upregulation. The overexpression of PKM2 sensitized HCC to treatment by immune checkpoint blockades, which was shown to lead to enhanced IFN-γ positive CD8 T cells in experimental HCC mouse models (Li et al.).

It has recently emerged that immune cells can have unique metabolic characteristics that also affect their effector function (8). The effect of metabolic changes on the activity of dendritic cells (DCs) is potentially an important topic that has not been widely addressed in the literature. The characteristics of DCs’ glucose metabolism, lipid metabolism, and amino acid metabolism, were addressed by Peng et al. They summarized what is known concerning the effect of the tumor microenvironment on DC metabolism and effector function as it relates to anti-tumor immunotherapy (Peng et al.).

## Conclusions

This special issue sought to highlight state of the art knowledge and discuss emerging biology associated with metabolic alterations and immune evasion in cancer metastasis. An expanded understanding of this complex and diverse field is uncovering exciting new possibilities for the targeted treatment of metastatic disease by effectively linking tumor immunity and cellular metabolism.

## Author Contributions

All authors listed have made a substantial, direct and intellectual contribution to the work, and approved it for publication.

## Conflict of Interest

The authors declare that the research was conducted in the absence of any commercial or financial relationships that could be construed as a potential conflict of interest.

## Publisher’s Note

All claims expressed in this article are solely those of the authors and do not necessarily represent those of their affiliated organizations, or those of the publisher, the editors and the reviewers. Any product that may be evaluated in this article, or claim that may be made by its manufacturer, is not guaranteed or endorsed by the publisher.
